# Supplementary material for: Versatile and sensitive detection of mono- and poly(ADP-ribosyl)ation reveals XRCC1-dependent remodelling of PARP1 signalling
Source: Nat Commun. 2026 Apr 2;17:3216. doi: 10.1038/s41467-026-71311-4 (PMC13057200; doi:10.1038/s41467-026-71311-4)
Supplement: Supplementary file 1 — Supplementary Information [file 41467_2026_71311_MOESM1_ESM.pdf]

SUPPLEMENTARY INFORMATION

Versatile and sensitive detection of mono- and poly(ADP-ribosyl)ation reveals XRCC1-dependent remodelling of PARP1 signalling

Helen Dauben, Mihaela Mihaljević, Andreas Kolvenbach, Maria Dilia Palumbieri, Chrysi Kapsali, Ina Huppertz and Ivan Matić

Supplementary Figures 1-7

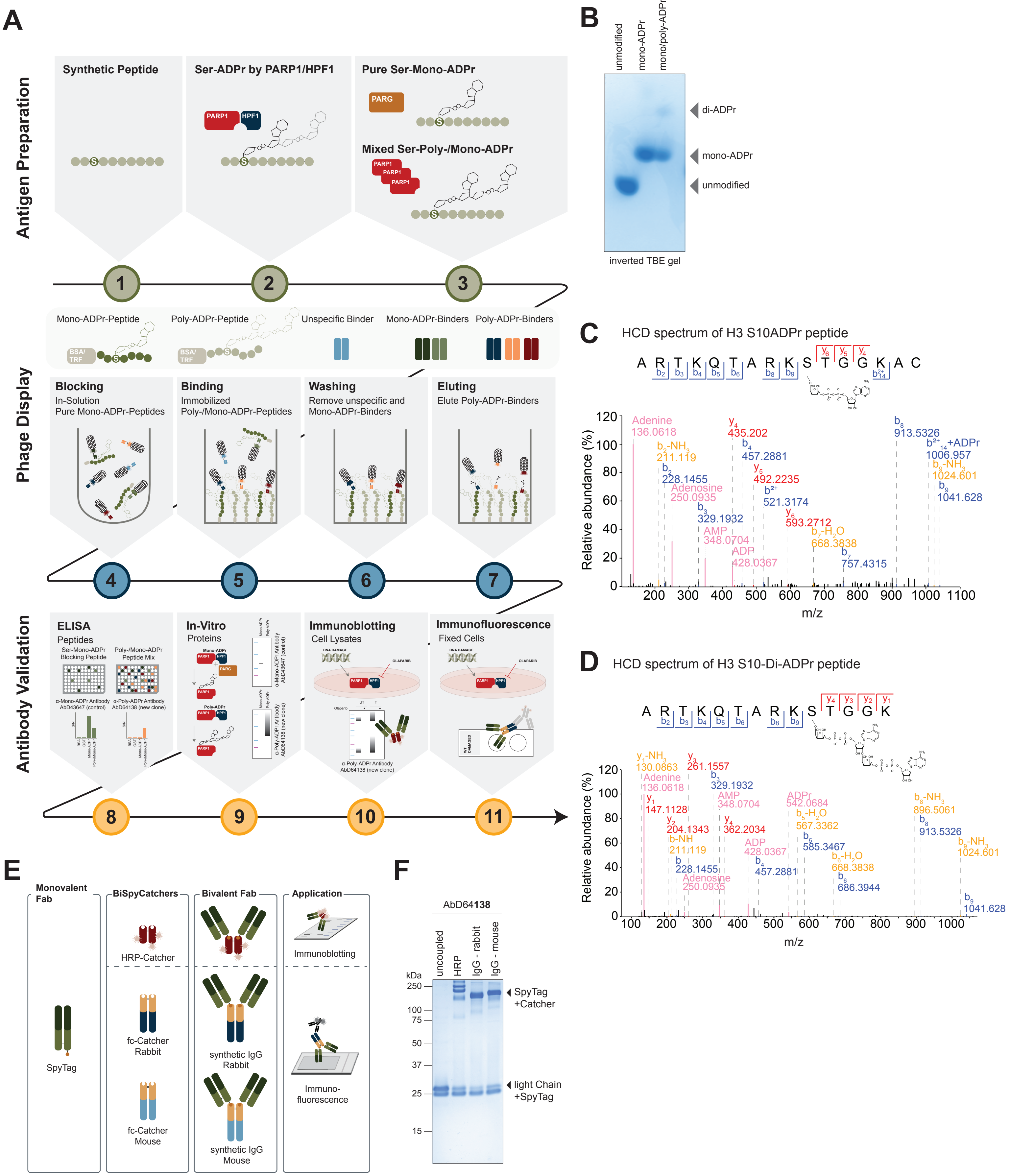
A
Antigen Preparation
Synthetic Peptide
Ser-ADPr by PARP1/HPF1
Pure Ser-Mono-ADPr
Mixed Ser-Poly-/Mono-ADPr
1
2
3
Mono-ADPr-Peptide
Poly-ADPr-Peptide
Unspecific Binder
Mono-ADPr-Binders
Poly-ADPr-Binders
Phage Display
Blocking
In-Solution Pure Mono-ADPr-Peptides
Binding
Immobilized Poly-/Mono-ADPr-Peptides
Washing
Remove unspecific and Mono-ADPr-Binders
Eluting
Elute Poly-ADPr-Binders
4
5
6
7
Antibody Validation
ELISA
Peptides
In-Vitro
Proteins
Immunoblotting
Cell Lysates
Immunofluorescence
Fixed Cells
8
9
10
11
B
unmodified
mono-ADPr
mono/poly-ADPr
di-ADPr
mono-ADPr
unmodified
inverted TBE gel
C
HCD spectrum of H3 S10ADPr peptide
A R T K Q T A R K S T G G K A C
Relative abundance (%)
m/z
D
HCD spectrum of H3 S10-Di-ADPr peptide
A R T K Q T A R K S T G G K
Relative abundance (%)
m/z
E
Monovalent Fab
BiSpyCatchers
Bivalent Fab
Application
HRP-Catcher
SpyTag
fc-Catcher Rabbit
fc-Catcher Mouse
synthetic IgG Rabbit
synthetic IgG Mouse
Immunoblotting
Immuno-fluorescence
F
AbD64138
uncoupled
HRP
IgG - rabbit
IgG - mouse
kDa
250
100
75
50
37
25
15
SpyTag +Catcher
light Chain +SpyTag

Supplementary Figure 1: (A) Detailed Schematic Illustration of Peptide Modification, Phage Display and Antibody Validation Process. Illustrations generated in Adobe Illustrator. Schematics adapted from Dauben et al. (2023). **(B)** Inverted-polarity TBE gel showing the unmodified peptide, mono-ADP-ribosylated peptide for blocking and mono-/poly-ADP-ribosylated peptide mix as antigen. **(C)** HCD spectrum of in vitro modified mono-ADP-ribosylated H3 serine 10 peptide, displaying mono-ADPr specific diagnostic ions (pink). N=1. **(D)** HCD spectrum of in vitro modified H3 carrying a di-ADPr on H3 as indicated by the mass of the precursor ion. Conventional mono-ADPr diagnostic ions were detected in this spectrum as well (pink). N=1. **(E)** Schematic Illustration of SpyTag Antibody Coupling options. Illustrations generated in Adobe Illustrator. Schematics adapted from Dauben et al. (2023). **(F)** Coomassie gel showing uncoupled, and coupled SpyTag antibodies with different SpyCatchers. Source data are provided as a Source Data file.

A

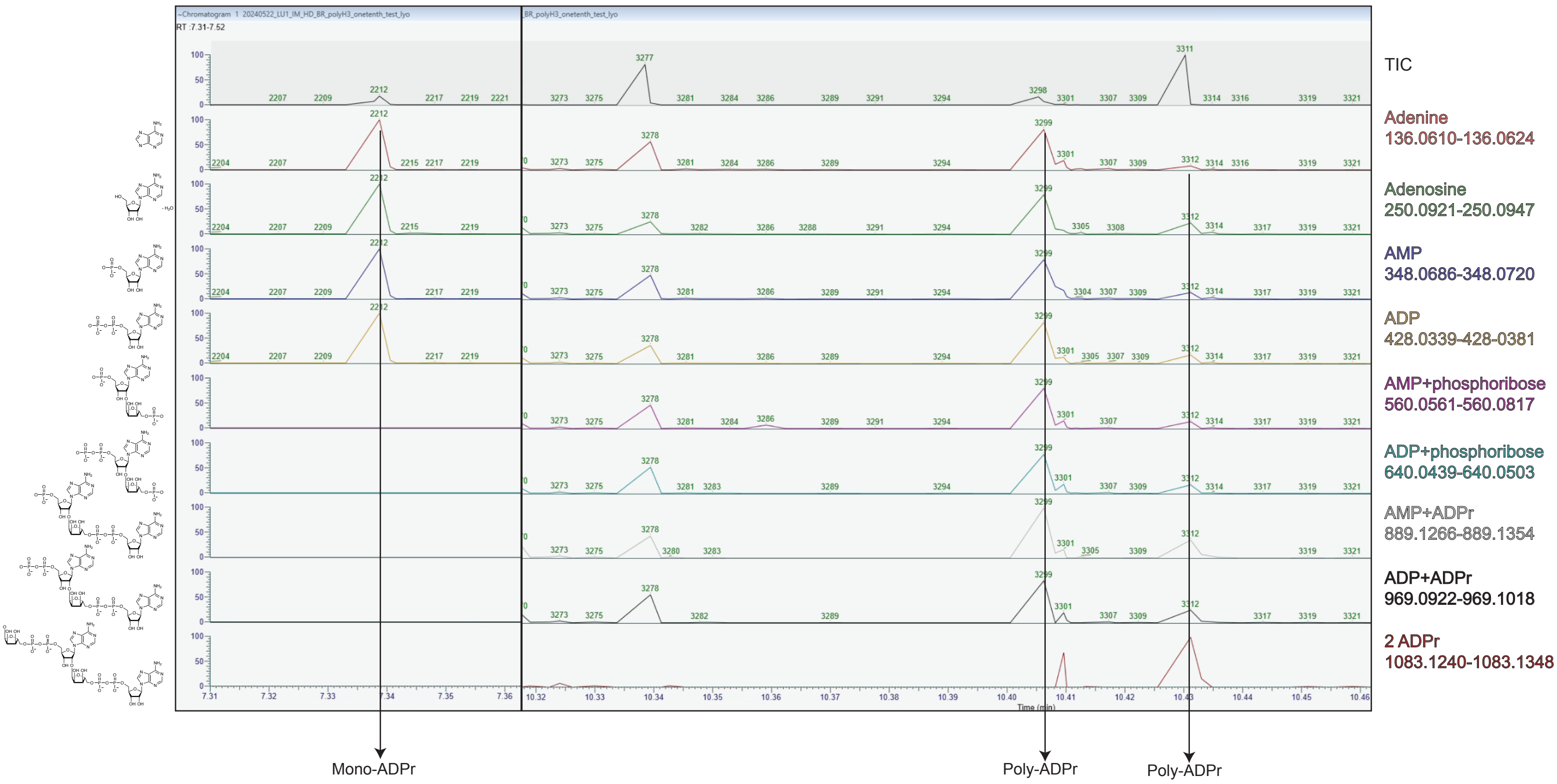

B

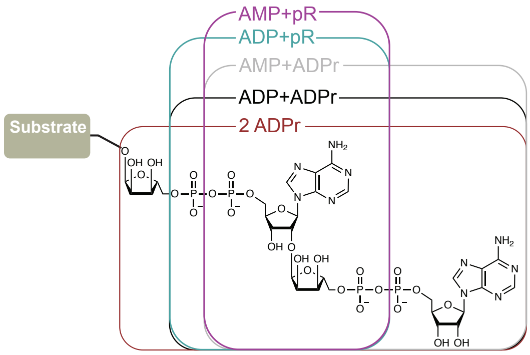

Diagnostic Ion	Chemical Composition	Neutral Mass	M+H+
Adenine	$C_5H_5N_5$	135.0545	136.0617
Adenosine - H_2O	$C_{10}H_{11}N_5O_3$	249.0861	250.0934
AMP	$C_{10}H_{14}N_5O_7P$	347.063	348.0703
ADP	$C_{10}H_{15}N_5O_{10}P_2$	427.0294	428.0366
ADPr	$C_{15}H_{21}N_5O_{13}P_2$	541.0611	542.0683
AMP+phosphoribose	$C_{15}H_{23}N_5O_{14}P_2$	559.0716	560.0789
ADP+phosphoribose	$C_{15}H_{24}N_5O_{17}P_3$	639.038	640.0453
AMP+ADPr	$C_{25}H_{35}N_{10}O_{20}P_3$	888.1242	889.1315
ADP+ADPr	$C_{25}H_{36}N_{10}O_{23}P_4$	968.1241	969.0978
2 ADPr	$C_{30}H_{42}N_{10}O_{26}P_4$	1082.1222	1083.1295

C

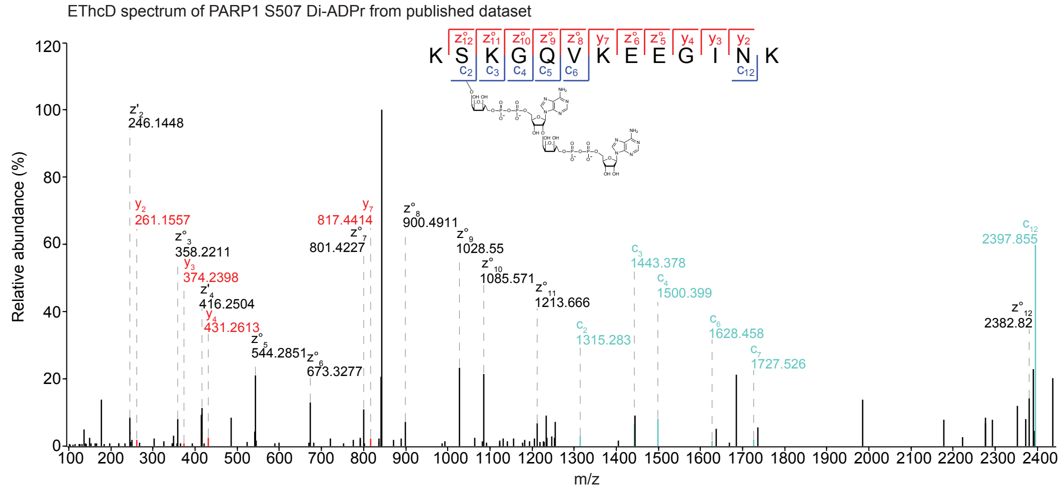

Supplementary Figure 2: Diagnostic Ions of poly-ADPr and identification of di-ADPr on S507 of PARP1. (A) Freestyle layout comparing the sets of diagnostic ions for mono-ADPr and poly-ADPr. The layout shows that in mono-ADPr containing spectra only Adenine, AMP and ADP are present. In contrast, in poly-ADPr spectra additional ions of poly-ADPr corresponding to AMP+phosphoribose (AMP+pR), ADP+phosphoribose (ADP+pR), AMP+ADPr, ADP+ADPr and Di-ADPr are present. The picture combines two screenshots of different regions of the same raw file stated in the top left corner. The screenshots are separated by the black line. **(B)** Table of diagnostic ions for poly-ADPr, explaining the different chemical compositions, the neutral masses and charged masses (M+H+) of poly-ADPr diagnostic ions. The poly-ADPr specific ions are highlighted in grey. **(C)** EThcD spectrum of PARP1 modified with di-ADPr on serine 507 from the analysed public dataset.

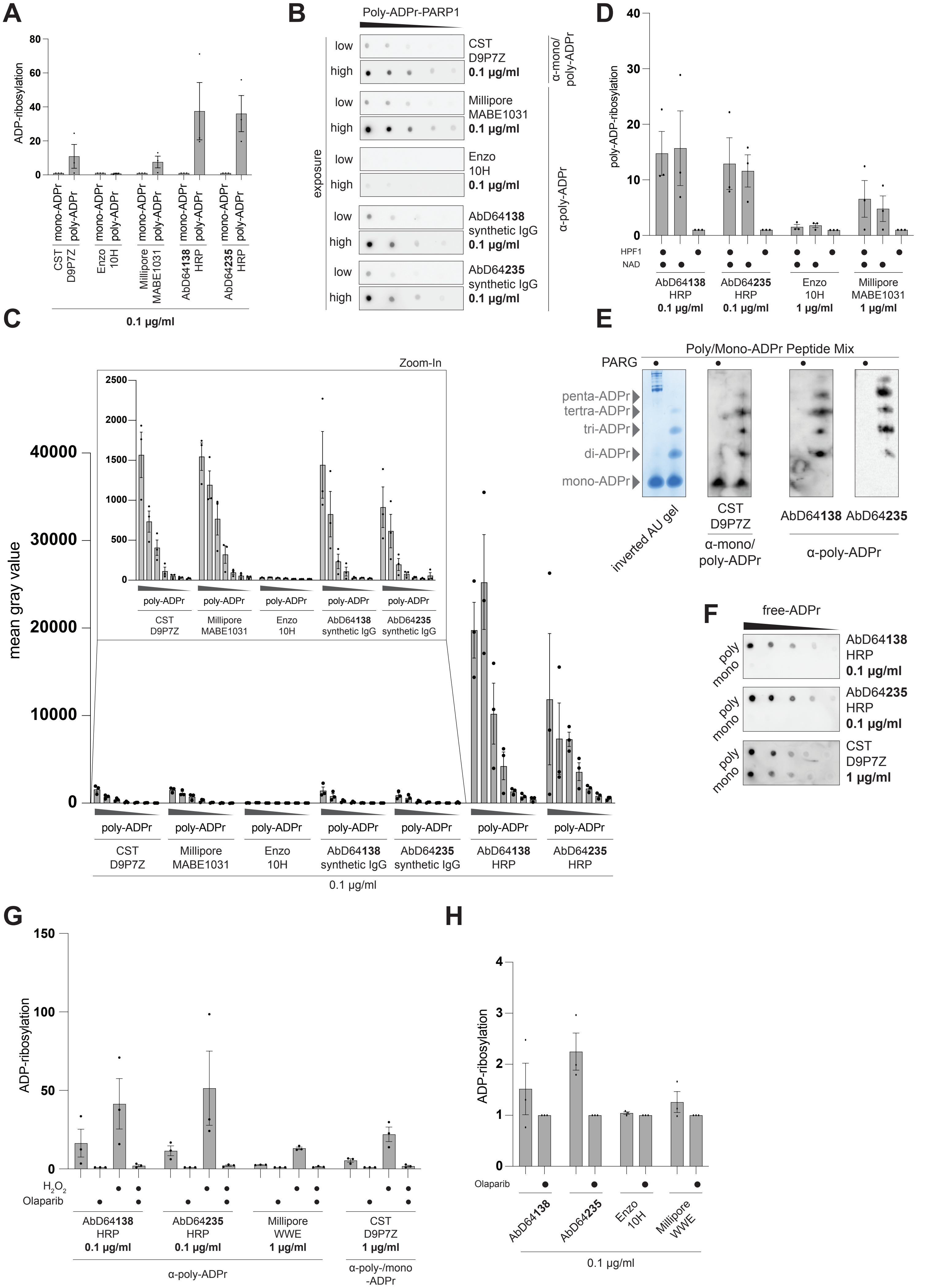
A
ADP-ribosylation
mono-ADPr
poly-ADPr
CST D9P7Z
Enzo 10H
Millipore MABE1031
AbD64138 HRP
AbD64235 HRP
0.1 µg/ml
B
Poly-ADPr-PARP1
exposure
low
high
CST D9P7Z 0.1 µg/ml
Millipore MABE1031 0.1 µg/ml
Enzo 10H 0.1 µg/ml
AbD64138 synthetic IgG 0.1 µg/ml
AbD64235 synthetic IgG 0.1 µg/ml
α-mono/ poly-ADPr
α-poly-ADPr
D
poly-ADP-ribosylation
HPF1
NAD
AbD64138 HRP 0.1 µg/ml
AbD64235 HRP 0.1 µg/ml
Enzo 10H 1 µg/ml
Millipore MABE1031 1 µg/ml
C
mean gray value
Zoom-In
poly-ADPr
CST D9P7Z
Millipore MABE1031
Enzo 10H
AbD64138 synthetic IgG
AbD64235 synthetic IgG
AbD64138 HRP
AbD64235 HRP
0.1 µg/ml
E
Poly/Mono-ADPr Peptide Mix
PARG
penta-ADPr
tertra-ADPr
tri-ADPr
di-ADPr
mono-ADPr
inverted AU gel
CST D9P7Z
α-mono/ poly-ADPr
AbD64138
AbD64235
α-poly-ADPr
F
free-ADPr
poly
mono
AbD64138 HRP 0.1 µg/ml
AbD64235 HRP 0.1 µg/ml
CST D9P7Z 1 µg/ml
G
ADP-ribosylation
H2O2
Olaparib
AbD64138 HRP 0.1 µg/ml
AbD64235 HRP 0.1 µg/ml
Millipore WWE 1 µg/ml
CST D9P7Z 1 µg/ml
α-poly-ADPr
α-poly-/mono -ADPr
H
ADP-ribosylation
Olaparib
AbD64138
AbD64235
Enzo 10H
Millipore WWE
0.1 µg/ml

Supplementary Figure 3: (A) Quantification of *in vitro* ADP-ribosylated PARP1 with (mono-ADPr) or without (poly-ADPr) PARG using Poly-/ Pan-/ Mono-ADPr antibodies. Combined Analysis of 3 Biological Replicates. Values are normalized to mono-ADPr conditions. Error bars represent SEM. **(B)** Additional Dot-blot analysis of *in vitro* auto-poly-ADPr of PARP1 in 1:2 dilution series comparing IgG formats. Detection was done in parallel resulting in comparable signal intensities. N = 3. **(C)** Quantification of Dot-blot analysis of *in vitro* auto-poly-ADPr of PARP1 in 1:2 dilution series using Poly-/ Pan-ADPr antibodies. Combined Analysis of 3 Biological Replicates. Here mean gray values are presented without normalization, after background subtraction. Error bars represent SEM**. (D)** Quantification of Immunoblot analysis of *in vitro* ADP-ribosylated PARP1 with (Ser-ADPr) or without (Asp/Glut-ADPr) HPF1 using Poly-ADPr antibodies. Combined Analysis of 3 Biological Replicates. Values are normalized to no NAD conditions. Error bars represent SEM. **(E)** Inverted-polarity Acid-Urea gel showing the mono-ADP-ribosylated peptide (+PARG) and mono-/poly-ADP-ribosylated peptide mix. Imperial Blue staining of Gel (right) and Immunoblot analysis (left). Detection was done on individual basis, signal intensities between different antibodies are not comparable. **(F)** Dot blot Analysis of free-ADPr (biotin linked) binding. Decreasing amounts of free mono- and poly-ADPr were detected on membrane using the indicated antibodies. Detection was done in parallel resulting in comparable signal intensities. **(G)** Quantification of Immunoblot analysis of SDS cell extracts from 2 mM H_2O_2-treated wild-type (WT) U2OS cells with and without 1 µM Olaparib treatment. Combined Analysis of 3 Biological Replicates. Values are normalized to only Olaparib conditions. Error bars represent SEM. **(H)** Quantification of Immunoblot analysis of SDS cell extracts from untreated wild-type (WT) hTERT RPE1 cells with and without 1 µM for 1 h Olaparib treatment. Combined Analysis of 3 Biological Replicates. Values are normalized to only Olaparib conditions. Error bars represent SEM. Source data are provided as a Source Data file.

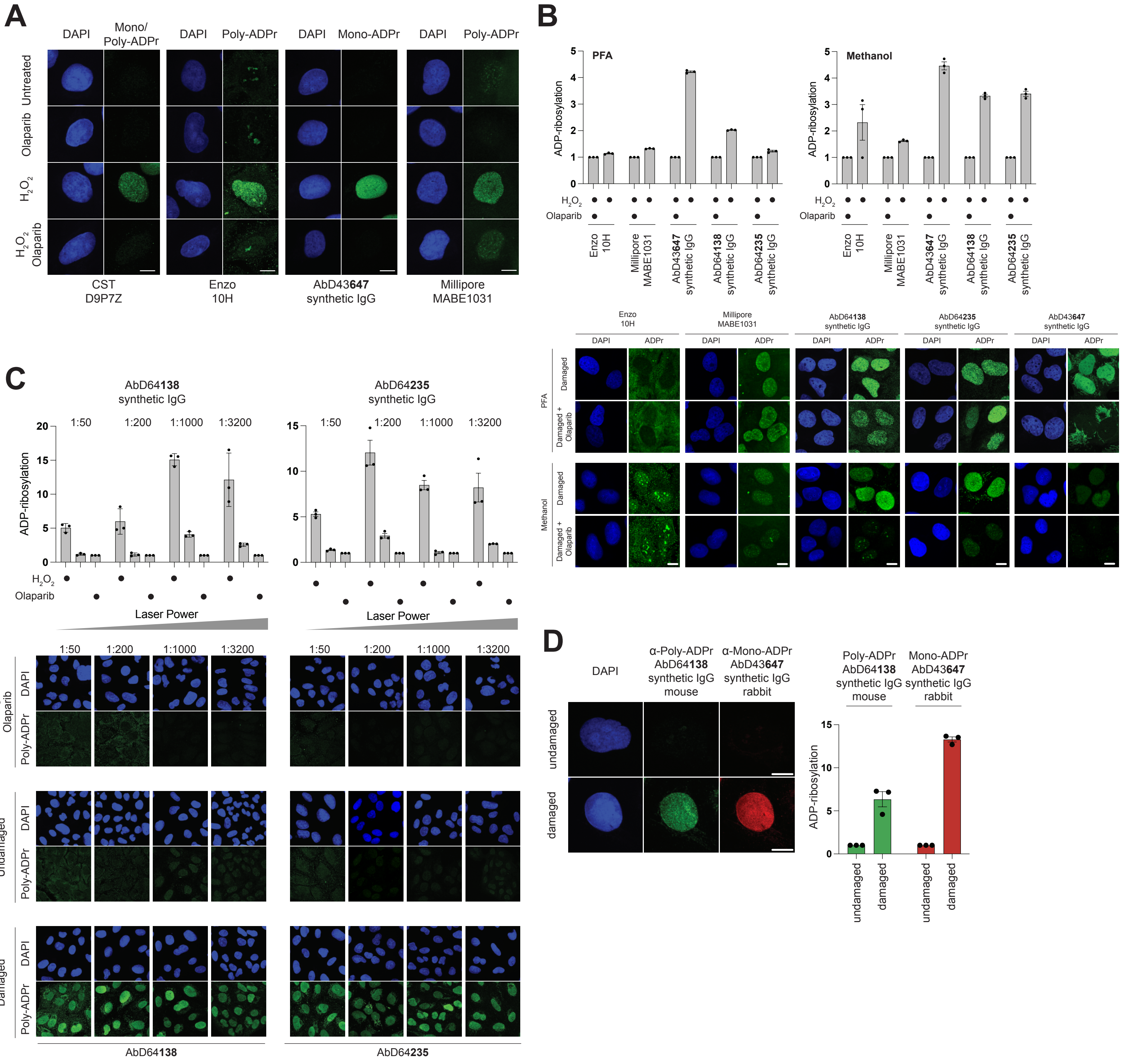
A
DAPI
Mono/ Poly-ADPr
Poly-ADPr
Mono-ADPr
Untreated
Olaparib
H2O2
H2O2 Olaparib
CST D9P7Z
Enzo 10H
AbD43647 synthetic IgG
Millipore MABE1031
B
PFA
Methanol
ADP-ribosylation
H2O2
Olaparib
Enzo 10H
Millipore MABE1031
AbD43647 synthetic IgG
AbD64138 synthetic IgG
AbD64235 synthetic IgG
ADPr
Damaged
Damaged + Olaparib
C
AbD64138 synthetic IgG
AbD64235 synthetic IgG
1:50
1:200
1:1000
1:3200
Laser Power
Undamaged + Olaparib
Undamaged
Damaged
AbD64138
AbD64235
D
α-Poly-ADPr AbD64138 synthetic IgG mouse
α-Mono-ADPr AbD43647 synthetic IgG rabbit
Poly-ADPr AbD64138 synthetic IgG mouse
Mono-ADPr AbD43647 synthetic IgG rabbit
undamaged
damaged

Supplementary Figure 4: (A) Immunofluorescent staining of mono-/ poly-/ pan-ADPr in 2 mM H_2O_2-treated WT U2OS cells with and without 1 µM Olaparib treatment using the indicated antibodies. Detection was done on individual basis, signal intensities between different antibodies are not comparable. Complementary representative Images from 1 Biological Replicate for Fig. 3F. Scale bar, 10 µM. **(B)** Immunofluorescent staining of mono-/ poly-ADPr in 2 mM H_2O_2-treated WT U2OS cells with and without 1 uM Olaparib treatment using the indicated antibodies. Fixated either for 15 min with 4% PFA or for 20 min with 100% Methanol. Detection was done on individual basis, signal intensities between different antibodies are not comparable. Signals are normalized to WT Olaparib conditions. Error bars represent SEM. Combined Analysis of 3 Biological Replicates. Representative Images from one Biological Replicate. Scale bar, 10 µM. **(C)** Immunofluorescent staining of poly-ADPr in 2 mM H_2O_2-treated WT U2OS cells with and without 1 uM Olaparib treatment using the indicated antibodies in different dilutions. Fixated with 100% Methanol. Detection was done on individual basis; signal intensities are not comparable. Signals are normalized to WT Olaparib only conditions. Error bars represent SEM. Combined Analysis of 3 Biological Replicates. Representative Images from one Biological Replicate. Scale bar, 10 µM. **(D)** Immunofluorescent Co-staining of poly- and mono-ADPr in 2 mM 10 min H_2O_2-treated WT U2OS cells using the indicated antibodies. Detection was done on individual basis, signal intensities between different antibodies are not comparable. Signals are normalized to WT undamaged conditions. Error bars represent SEM. Combined Analysis of 3 Biological Replicates. Representative Images from 1 Biological Replicate. Scale bar, 10 µM. Source data are provided as a Source Data file.

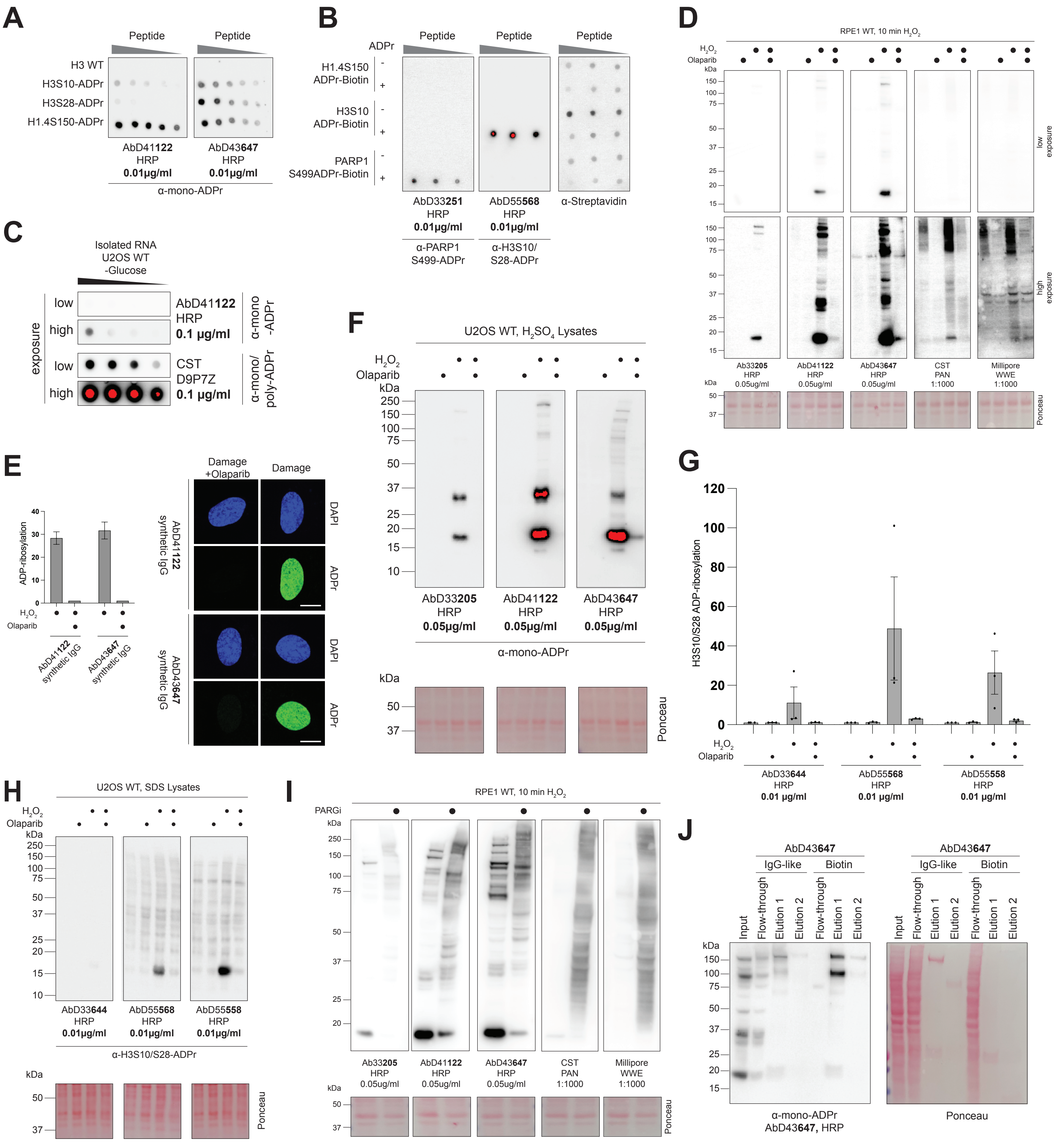

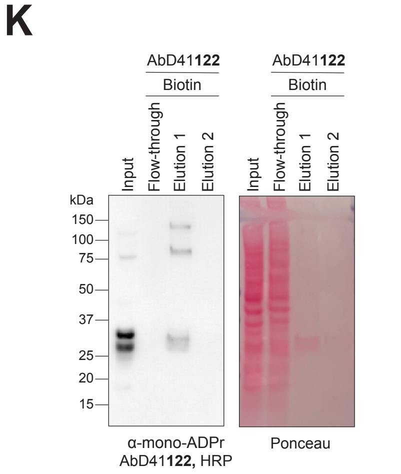

L

Name	Specificity	Tested Appli.	Recom. Dilution WB (µg/ml)	Source	Availability
AbD33**204**	α-mono-ADPr	WB, IF, IP	1-2	Bonfiglio, et al. Cell. 2020	Commercialised with BioRad
AbD43**647**	α-mono-ADPr	WB, IF, IP	0.05-0.1	Longarini, et al. Mol Cell. 2023	Commercialised with BioRad
AbD33**205**	α-mono-ADPr	WB, IF, IP	1-2	Bonfiglio, et al. Cell. 2020	Commercialised with BioRad
AbD41**122**	α-mono-ADPr	WB, IF, IP	0.05-0.1	This Manuscript	Upon Request, Commercialisation planned
AbD33**644**	α-H3S10/S28-ADPr	WB	0.4	Bonfiglio, et al. Cell. 2020	Commercialised with BioRad
AbD55**558**	α-H3S10/S28-ADPr	WB	0.01-0.05	This Manuscript	Upon Request
AbD55**568**	α-H3S10/S28-ADPr	WB	0.01-0.05	This Manuscript	Upon Request,Commercialisation planned
AbD33**251**	α-PARP1S499-ADPr	WB	0.4	Bonfiglio, et al. Cell. 2020	Commercialised with BioRad
AbD33**641**	α-Pan-ADPr	WB, IF	1-2	Bonfiglio, et al. Cell. 2020	Commercialised with BioRad
AbD64**138**	α-Poly-ADPr	WB, IF, IP	0.05-0.1	This Manuscript	Upon Request, Commercialisation planned
AbD64**235**	α-Poly-ADPr	WB, IF, IP	0.05-0.1	This Manuscript	Upon Request

Supplementary Figure 5: **(A)** Dot-blot analysis of *in vitro* modified mono-ADPr peptide dilution series using Mono-ADPr. N = 3. **(B)** Dot-blot analysis of *in vitro* modified mono-ADPr peptide dilution series using Site-Specific-ADPr antibodies. N = 2. **(C)** Dot Blot analysis of isolated RNA from U2OS cells after glucose starvation. Detection was done in parallel resulting in comparable signal intensities. N = 3. **(D)** Immunoblot analysis of SDS cell extracts from 150 µM H_2O_2-treated wild-type (WT) hTERT RPE1 cells with and without 1 uM Olaparib treatment with the indicated antibodies. Detection was done in parallel resulting in comparable signal intensities. N = 3. **(E)** Immunofluorescent staining of mono -ADPr in 150 µM H_2O_2-treated WT hTERT RPE1 cells with and without 1 uM Olaparib treatment using the indicated antibodies. Detection was done in parallel resulting in comparable signal intensities. Signals are normalized to WT Olaparib conditions. Error bars represent SEM. Combined Analysis of 3 Biological Replicates. Representative Images from 1 Biological Replicate. Scale bar, 10 µM. **(F)** Immunoblot analysis of H_2SO_4 cell extracts from 2 mM H_2O_2-treated wild-type (WT) U2OS cells with and without 1 uM Olaparib treatment with mono-ADPr antibodies. Detection was done in parallel resulting in comparable signal intensities. Red color represents saturated signal. N = 3. **(G)** Quantification of Immunoblot analysis of H_2SO_4 cell extracts from 2 mM H_2O_2-treated wild-type (WT) U2OS cells with and without 1 µM Olaparib treatment with H3S10/S28-ADPr site-specific antibodies. Combined Analysis of 3 Biological Replicates. Values are normalized to untreated conditions. Error bars represent SEM. **(H)** Immunoblot analysis of SDS cell extracts from 2 mM H_2O_2-treated wild-type (WT) U2OS cells with and without 1 uM Olaparib treatment with H3S10/S28-ADPr Site-Specific antibodies. Detection was done in parallel resulting in comparable signal intensities. N = 3. **(I)** Immunoblot analysis of SDS cell extracts from 150 µM H_2O_2-treated wild-type (WT) hTERT RPE1 cells with and without 1 uM PARG inhibition treatment with the indicated antibodies. Detection was done in parallel resulting in comparable signal intensities. N = 3. **(J)** Immunoprecipitation of mono-ADPr using AbD43647 clone in IgG or Biotin coupled format with Protein A or Streptavidin agarose beads. Immunoblot detection performed with AbD43647 HRP coupled. N = 3. **(K)** Immunoprecipitation of mono-ADPr using AbD41122 clone in Biotin coupled format with Streptavidin agarose beads. Immunoblot detection performed with AbD41122 HRP coupled. N = 3. **(L)** Overview of Antibody based ADPr-Toolbox generated in our lab, with recommended applications and dilutions. Dilutions for immunoblotting refer to strong signals with induced DNA damage and might be adapted according to expected signal intensities. Source data are provided as a Source Data file.

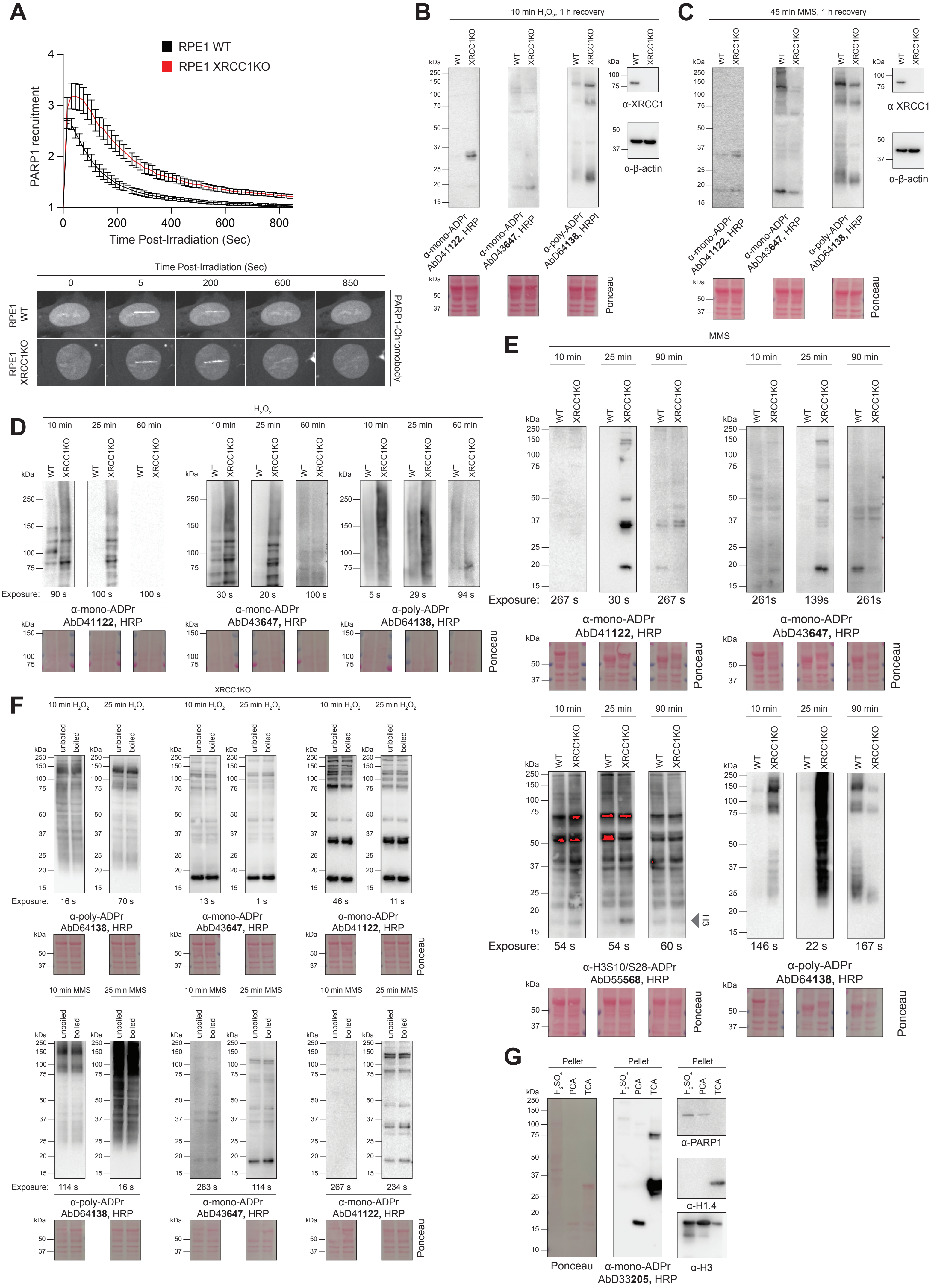
A
RPE1 WT
RPE1 XRCC1KO
PARP1 recruitment
Time Post-Irradiation (Sec)
0
200
400
600
800
Time Post-Irradiation (Sec)
0
5
200
600
850
RPE1 WT
RPE1 XRCC1KO
PARP1-Chromobody
B
10 min H2O2, 1 h recovery
WT
XRCC1KO
α-XRCC1
α-β-actin
α-mono-ADPr AbD41122, HRP
α-mono-ADPr AbD43647, HRP
α-poly-ADPr AbD64138, HRP
Ponceau
C
45 min MMS, 1 h recovery
D
H2O2
10 min
25 min
60 min
Exposure:
90 s
100 s
100 s
30 s
20 s
100 s
5 s
29 s
94 s
E
MMS
10 min
25 min
90 min
Exposure:
267 s
30 s
267 s
261s
139s
261s
54 s
54 s
60 s
146 s
22 s
167 s
H3
α-H3S10/S28-ADPr AbD55568, HRP
F
XRCC1KO
10 min H2O2
25 min H2O2
10 min MMS
25 min MMS
unboiled
boiled
Exposure:
16 s
70 s
13 s
1 s
46 s
11 s
114 s
16 s
283 s
114 s
267 s
234 s
G
Pellet
H2SO4
PCA
TCA
α-PARP1
α-H1.4
α-H3
α-mono-ADPr AbD33205, HRP
kDa

Supplementary Figure 6: (A) Real-time live-cell detection of PARP1 recruitment in hTERT RPE1 WT or XRCC1KO cells. Representative recruitment kinetics (top) and confocal images (bottom). Error bars represent SEM. Scale bar, 10 µM. N = 3. **(B)** Immunoblot analysis of SDS cell extracts from 10 min 150 µM H_2O_2-treated followed by 1 h drug free medium recovered wild-type (WT) and XRCC1KO hTERT RPE1 cells with the indicated antibodies. N = 5. **(C)** Immunoblot analysis of SDS cell extracts from 45 min 1.8 mM MMS-treated followed by 1 h drug free medium recovered wild-type (WT) and XRCC1KO hTERT RPE1 cells with the indicated antibodies. N = 3. **(D)** High MW Immunoblot analysis of SDS cell extracts from continuously 150 µM H_2O_2-treated wild-type (WT) and XRCC1KO hTERT RPE1 cells for the indicated time points with the indicated antibodies. Using 3-8 % Tris Acetate Gel and Tris/Tricine/SDS running buffer. N = 3. **(E)** Immunoblot analysis of SDS cell extracts from continuously 1.8 mM MMS H_2O_2-treated wild-type (WT) and XRCC1KO hTERT RPE1 cells for the indicated time points with the indicated antibodies. Red color represents saturated signal. Time points are shown in separate panels because different exposure times were required to accurately capture signal intensities, as indicated. N = 3. **(F)** Immunoblot analysis of SDS cell extracts from continuously 150 µM H_2O_2-treated XRCC1KO hTERT RPE1 cells for the indicated time points with the indicated antibodies unboiled or boiled for 5 min at 95 °C before loading. Time points are shown in separate panels because different exposure times were required to accurately capture signal intensities, as indicated. **(G)** Immunoblot analysis of three step Histone H1 isolation, running samples of each pellet with indicated antibodies. N = 3. Source data are provided as a Source Data file.

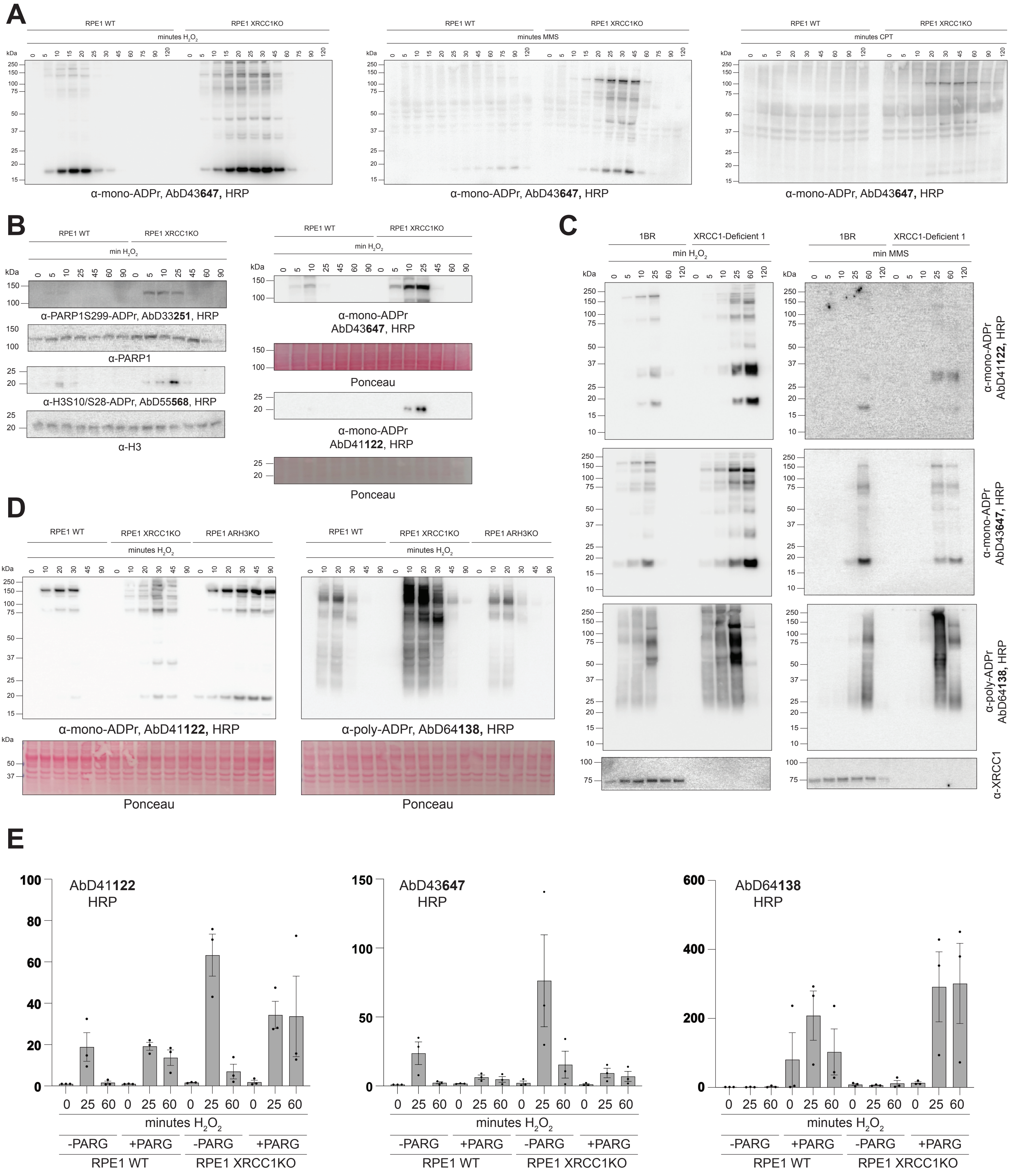
A
RPE1 WT
RPE1 XRCC1KO
minutes H2O2
α-mono-ADPr, AbD43647, HRP
minutes MMS
minutes CPT
B
min H2O2
α-PARP1S299-ADPr, AbD33251, HRP
α-PARP1
α-H3S10/S28-ADPr, AbD55568, HRP
α-H3
α-mono-ADPr
AbD43647, HRP
Ponceau
AbD41122, HRP
C
1BR
XRCC1-Deficient 1
min MMS
α-mono-ADPr AbD41122, HRP
α-mono-ADPr AbD43647, HRP
α-poly-ADPr AbD64138, HRP
α-XRCC1
D
RPE1 ARH3KO
α-mono-ADPr, AbD41122, HRP
α-poly-ADPr, AbD64138, HRP
E
AbD41122 HRP
AbD43647 HRP
AbD64138 HRP
-PARG
+PARG

Supplementary Figure 7: (A) Additional Immunoblot analysis of SDS cell extracts from continuously 150 µM H_2O_2, 1.8 mM MMS or 15 µM CPT-treated wild-type (WT) and XRCC1KO hTERT RPE1 cells for the indicated time points with the indicated antibodies. N = 3. **(B)** Immunoblot analysis of SDS cell extracts from continuously 150 µM H_2O_2-treated wild-type (WT) and XRCC1KO hTERT RPE1 cells for the indicated time points with site-specific ADPr-Antibodies. N = 3. **(C)** Immunoblot analysis of SDS cell extracts from continuously 150 µM H_2O_2- or 1.8 mM MMS-treated primary fibroblasts from healthy patients (1BR) or XRCC1 deficient patients (XD1) for the indicated time points with the indicated antibodies. N = 3. **(D)** Immunoblot analysis of SDS cell extracts from continuously 150 µM H_2O_2-treated wild-type (WT), XRCC1KO or ARH3KO hTERT RPE1 cells for the indicated time points with indicated Antibodies. N = 3. **(E)** Quantification of Immunoblot analysis of SDS cell extracts from 150 µM H_2O_2-treated wild-type (WT) and XRCC1KO hTERT RPE1 cells with and without 1 µM PARG inhibition for the indicated time points. Combined Analysis of 3 Biological Replicates. Values are normalized to untreated (0 min, no PARG, WT) conditions. Error bars represent SEM. Source data are provided as a Source Data file.
