# Supplementary material for: Versatile and sensitive detection of mono- and poly(ADP-ribosyl)ation reveals XRCC1-dependent remodelling of PARP1 signalling
Source: Nat Commun. 2026 Apr 2;17:3216. doi: 10.1038/s41467-026-71311-4 (PMC13057200; doi:10.1038/s41467-026-71311-4)
Supplement: Supplementary file 2 — Description of Additional Supplementary Files [file 41467_2026_71311_MOESM2_ESM.pdf]

Description of Additional Supplementary Files

File name: Supplementary Data 1

Description: The file contains the di-ADPr sites output of MaxQuant analysis, as reported in Figure 2B, C and D as well as in Supplementary Figure 2C. Identified sites with localization probability > 80 %, score > 40 and delta score > 20 are reported. Additionally, the dataset lists the published raw data used for the reanalysis.
